# Cell Calcium Imaging as a Reliable Method to Study Neuron–Glial Circuits

**DOI:** 10.3389/fnins.2020.569361

**Published:** 2020-10-02

**Authors:** Ricardo Augusto de Melo Reis, Hércules Rezende Freitas, Fernando Garcia de Mello

**Affiliations:** ^1^Laboratório de Neuroquímica, Instituto de Biofísica Carlos Chagas Filho, CCS, Universidade Federal do Rio de Janeiro, Rio de Janeiro, Brazil; ^2^Department of Pathology and Laboratory Medicine, MIND Institute, University of California, Davis, Sacramento, CA, United States

**Keywords:** calcium imaging, neuron–glia, ATP, glioblastoma, fluorescent indicator

## Abstract

Complex dynamic cellular networks have been studied in physiological and pathological processes under the light of single-cell calcium imaging (SCCI), a method that correlates functional data based on calcium shifts operated by different intracellular and extracellular mechanisms integrated with their cell phenotypes. From the classic synaptic structure to tripartite astrocytic model or the recent quadripartite microglia added ensemble, as well as other physiological tissues, it is possible to follow how cells signal spatiotemporally to cellular patterns. This methodology has been used broadly due to the universal properties of calcium as a second messenger. In general, at least two types of receptor operate through calcium permeation: a fast-acting ionotropic receptor channel and a slow-activating metabotropic receptor, added to exchangers/transporters/pumps and intracellular Ca^2+^ release activated by messengers. These prototypes have gained an enormous amount of information in dynamic signaling circuits. SCCI has also been used as a method to associate phenotypic markers during development and stage transitions in progenitors, stem, vascular cells, neuro- and glioblasts, neurons, astrocytes, oligodendrocytes, and microglia that operate through ion channels, transporters, and receptors. Also, cancer cells or inducible cell lines from human organoids characterized by transition stages are currently being used to model diseases or reconfigure healthy cells in terms of the expression of calcium-binding/permeable molecules and shed light on therapy.

## Introduction

Since the revolutionary work of Camilo Golgi, Santiago Ramon y Cajal, and Charles Sherrington at the turn of the twentieth century, scientists have attempted to correlate structure and function to understand the nervous system. At that time, cells were labeled with the technique of silver impregnation, discovered by Golgi in 1873 and later applied by Cajal to almost every part of the brain, from 1887 until the rest of his life. At the same time (1882–1883), Sydney Ringer described the properties of a physiological solution acknowledging the role of Ca^2+^ (in addition to Na^+^ and K^+^) (Ringer and Buxton, 1887), essential to contraction and the observed variations of ventricular volume of excised frog heart (Miller, 2004). Around 1910, Sherrington conceptualized the synapse, only deeply comprehended in terms of active zones around the 1950s, with the development of the electronic microscope. Electron-dense pre-synaptic sites were characterized and shown to host multifunctional proteins, including calcium channels. In parallel, the work of brilliant electrophysiologists such as John Eccles, a defendant of the concept of electrical synapse, and the pillars of bioelectrogenesis and action potential, Alan Hodgkin, Andrew Huxley, Bernard Katz, Ricardo Miledi, among others, added another level of signaling in modern neurophysiology.

Cellular neuroscience emerged in the last half-century as a merge of anatomy, histology, physiology, neurochemistry, cell biology, and behavioral sciences to gain information on the rules of neural ensembles and behavior. Lately, questions evolved with data revealed from *in vitro* fluorescence and/or confocal microscope and/or *in vivo* non-invasive high-resolution imaging techniques as the two-photon and/or lightweight miniaturized microscopy that permit optical recordings in freely moving animals for weeks.

Cellular networks can now be visualized with powerful imaging methods using advanced statistics to allow dynamic molecular messages that drive structure and function with fluorescent shuffled reporters (Lichtman et al., 2008). Pharmacological approaches based on functional proteomics add several layers of complexity, evaluated through optogenetics (Kim et al., 2017) or in combination with electrical or optical recordings, providing powerful strategies to alter the neural function and gain behavioral data (Frackowiak and Markram, 2015). This will soon lead to further details on the plasticity and the adaptation of the brain to understand still intangible fields such as cognition and consciousness (Cook, 2008). Here, we will focus on the properties of Ca^2+^ signaling dynamics related to membrane proteins found virtually on every single neural cell, but due to limitation, it will be restricted to selective models.

**FIGURE 1:**
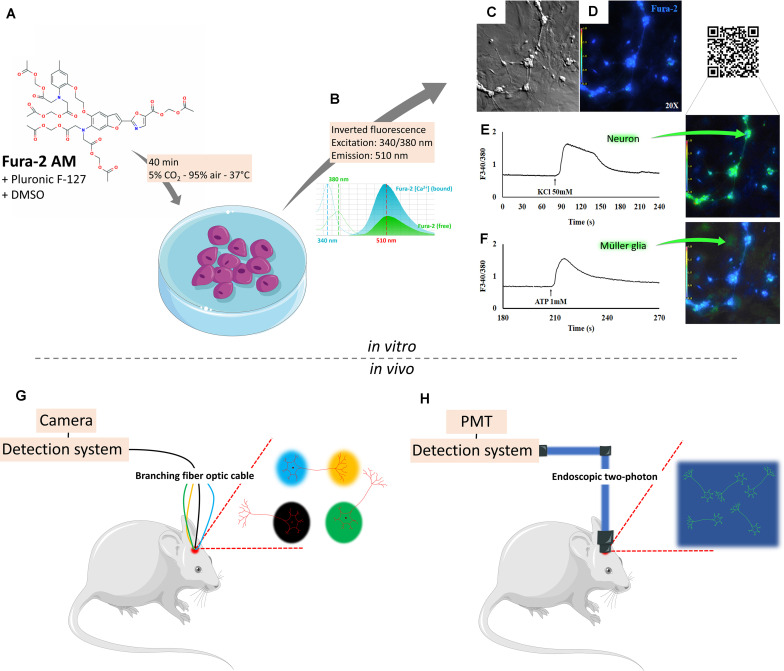
Detecting intracellular calcium changes through single-cell calcium imaging. Single-cell calcium imaging evaluates intracellular Ca^2+^ dynamics through calcium probes as Fura-2 AM **(A)**, a lipophilic acetate ester (AM) that permeates the cell membrane in an apolar environment (DMSO + pluronic F-127), allowing cleavage by intracellular esterases. These sensors show stronger fluorescence and better affinity for Ca^2+^ and selectivity against Mg^2+^ in the presence of Ca^2+^ shifts **(B)**. Neurons and glia in bright field **(C)** or under fluorescence **(D)** when activated by KCl 50 mM **(E)** or ATP 1 mM **(F)** show selective responses. In parallel, the use of inducible genetically encoded calcium indicators and a variety of detection systems made probing neural cell behavior in live animals a possibility. Through a photoreceiver, it is possible to detect changes in cell fluorescence as the animal is awake and freely behaving. An optimized version of this technique can be obtained through multi-site photometry **(G)**, which allows for the detection of spatiotemporal changes as the animal responds to varying environmental patterns. In this method, signals are detected by a complementary metal oxide semiconductor (CMOS) camera. Direct bidimensional fluorescence can be detected with the use of one-photon microendoscopes, capable of imaging a region of interest (ROI) inside the living brain, which in turn is detected by a CMOS camera. Two-photon endoscopes can be used to generate a high-fidelity view of a given ROI. In this case, images are generated after detection by photomultiplier tubes **(H)**.

Single-cell calcium imaging (SCCI) is a versatile methodology used to visualize calcium shifts, from extracellular or intracellular stores, associated with a profile of molecules selectively expressed on living individual cells. Calcium has a central role on almost every cellular task, as a messenger ion and/or interacting with a multitude of binding proteins, adjusting several activities, e.g., gene transcription, cell birth, proliferation, migration, signaling/excitation/contraction, cell growth, metabolism, cytoskeletal dynamics, differentiation, synaptic transmission and plasticity, survival, and death (Carafoli and Krebs, 2016; Islam, 2020). Calcium concentration in the extracellular space is ∼1.8–2.5 × 10^–3^ M, at least 2 × 10^4^ higher than in the cytosolic compartment (typical resting levels ∼1–2 × 10^–7^ M). The expression of calcium-permeable channels, transporters, receptors, and effector molecules is essential to understand spatiotemporal signaling in living cell compartments. Calcium imaging evaluates intracellular Ca^2+^ dynamics that originated with the development of calcium dyes such as 1,2-bis(o-aminophenoxy)ethane-N,N,N’,N’-tetraacetic acid from the laboratory of Roger Y. Tsien, with high selectivity against magnesium and protons (Tsien, 1980) and later improved as selective Ca^2+^ indicator, “quin2”, studied inside intact lymphocytes (Tsien et al., 1982). Lipophilic groups added [acetoxymethyl or acetate ester (AM) groups] to the charged indicators gave a membrane-permeant property and hidden charges, allowing cleavage by constitutively expressed intracellular esterases. These sensors show stronger fluorescence (intensity increases up to 30-fold) and better affinity for Ca^2+^ and selectivity against Mg^2+^ in the presence of Ca^2+^ shifts (Grynkiewicz et al., 1985). The first six dyes generated [from which fura-2 (Figure 1A) and indo-1 are the ratiometric] [measure free Ca^2+^ concentration with Ca^2+^-free and Ca^2+^-bound forms having two distinct peaks (Figure 1B)] prototypes, based on the fluorescence intensity detected on two wavelengths (Rudolf et al., 2003; Bruton et al., 2020), and are widely recognized to track calcium shifts within cells (Figures 1C–F), from intracellular or extracellular sources. The development of better fluorescent microscopes and computation methods yielded high-resolution optical data with a high degree of spatial and temporal resolution to image calcium signaling on living cells and organisms.

The use of genetically coded calcium indicators (GECI) allowed the study of cellular structure/function to match behavior responses. GECIs have been developed as fused proteins to monitor the spatiotemporal patterns of intracellular Ca^2+^ dynamics in functional compartments of living organisms. This strategy has been used from *Drosophila* to the intact brain of transgenic rodents and not only in the cytoplasmic environment but also in organelles such as the mitochondria or the endoplasmic reticulum. Excellent recent reviews are available, so we will not extend on this matter (Inoue, 2020). Briefly, to overcome obstacles in dye loading faced by organic indicators and other variabilities on the lack of cell-type selectivity, chimeric protein has been engineered and named “chameleons” (Miyawaki et al., 1997) based on the color change of fluorescent proteins fused to a Ca^2+^ binding protein (usually calmodulin or troponin C) and M13 [a peptide with a primary sequence based on the skeletal muscle myosin light chain kinase (residues 577–602). This peptide acts as a spacer and binds to Ca^2+^, modifying the conformation from a weak to strong fluorescence complex. Fluorescence resonance energy transfer is based on Ca^2+^ binding and conformation change of calmodulin around the M13 domain. As an example, GCaMP acts a high-affinity Ca^2+^ probe prototype composed of a single green fluorescent protein (Nakai et al., 2001). Variations (GCaMP3, GCaMP5, and GCaMP6) have been prepared, which are converted from green to red following exposure to blue-green light at 450–500 nm (Ai et al., 2015).

A current challenge faced with GECI live imaging is related to the identification of specific subsets of active neurons within a larger cell population. Accomplishing this task is important to understand ensemble responses and to improve the visualization of signal integration across multiple brain regions. Although several efforts were able to ensure a somewhat automated processing of these imaging results through classic machine learning techniques (Mukamel et al., 2009; Kaifosh et al., 2014; Guan et al., 2018), current approaches are now entering the realm of convolutional neural networks, which can be used in a 3D architecture to segment active neurons on different layers of the same imaging target (Soltanian-Zadeh et al., 2019). Also, accessible algorithms for imaging motion correction have been recently developed and can be used to improve the output quality of automated segmentation networks (Pnevmatikakis and Giovannucci, 2017).

It is interesting to notice that, while the analysis of live Ca^2+^ imaging data is becoming increasingly accessible and automated through a large array of online resources, the *in vivo* experiments demand progressively more sophisticated tools, either to improve field-of-view resolution or to increase the number of simultaneous detections from different brain regions. The use of confocal and multiphoton laser scanning microscopy helped to extend to the larger scale, obtaining data on organ systems, from small GECI-expressing transparent alive invertebrates to awake transgenic mammals (Russell, 2011). Through a photoreceiver, it is now possible to detect changes in cell fluorescence in different *in vivo* models, as imaging for somatosensorial or visual inputs inducing synaptic plasticity on the dendritic spine patterns on cortical neurons or calcium imaging in awake and freely behaving animals (Grienberger and Konnerth, 2012). One exciting possibility would be the integration of multiple neuroimaging methods, combining low spatiotemporal fidelity from functional magnetic resonance imaging (fMRI) with multiple-site photometry (Figure 1G). Several groups have reported simultaneous acquisition of blood oxygen level-dependent (BOLD) fMRI, regularly used for non-invasive functional neuroimaging, combined with chronically implanted optical fiber, which allow data acquisition of identified cells from transgenic models or through viral electroporation delivered with GCaMP6 (Schlegel et al., 2018), and understand the relationship of neural and vascular signals (Albers et al., 2018). The advantage of animals placed in an MRI scanner coupled to a fiber photometry through a silicon photo multimeter is the prevention of electromagnetic interference seen in electrophysiological recordings during MRI scanning (Logothetis et al., 2001). The increased popularity of this technique came with the development of the multi-channel fiber photometry system to simultaneously monitor neural activities in several brain areas of an animal or in different animals (Guo et al., 2015). It was initially prepared to explore this correlation in rat somatosensory cortex, where forepaw stimulation evoked fast calcium signals of neuronal origin (Schulz et al., 2012) or, as reported, slower calcium signals derived from astrocyte networks. Coupling of GCaMP6-based calcium signal to neural activity produces a novel opportunity with a reproducible temporal relationship, validated, for example, with a visual stimulation experiment, during which robust increases of both calcium and BOLD signals were observed in the superior colliculus of rats (Liang et al., 2017). A following investigation demonstrated that these signals emerge in a region-specific pattern from the SC and the lateral geniculate nucleus (Tong et al., 2019). This strategy has also permitted the mapping of neuronal circuits in the whole mouse cortex, yielding a connective map spanning several cortical subregions (Lake et al., 2018; Schlegel et al., 2018). Today it is possible to study anesthetized or freely behaving animals and probe calcium signals in a variety of brain regions during development, aging, or disease (Wright et al., 2017; Schlegel et al., 2018). An optimized version allows multi-fiber photometry and optogenetic perturbations across many regions in the mammalian brain (the authors managed to detect GCaMP6m-generated Ca^2+^ fluorescence from striatal, thalamic, hippocampal, and cortical areas). In this sense, the spatiotemporal changes of neuronal activity are registered as a go/no-go texture discrimination task runs on awake mice responding to varying environmental patterns (Sych et al., 2019). Alternatively, *in vivo* astrocytic microdomains show spatially restricted synchronized calcium transients that last a few seconds upon activation of transmitter receptors, observed in both anesthetized and awake animals [reviewed in Nimmerjahn and Bergles (2015)]. In this method, signals are detected by a complementary metal oxide semiconductor (CMOS) camera. Direct bidimensional fluorescence can be detected with the use of a one-photon microendoscope, capable of imaging a region of interest (ROI) inside the living brain, which in turn is detected by a CMOS camera. Two-photon endoscopes can be used to generate a high-fidelity view of a given ROI. In this case, images are generated after detection by photomultiplier tubes (Figure 1H). In relation to the astrocytic compartment, data on GCaMP-mediated Ca^2+^ optical fiber recordings described a brain-state dependency of both astrocytic Ca^2+^ and BOLD fMRI signals (Wang M. et al., 2018). Distinct Ca^2+^ signals combined with positive BOLD signals and intrinsic astrocytic Ca^2+^ signals coupled with negative BOLD signals were reported. Indeed recent data from anesthetized transgenic mice expressing G-CaMP7 in astrocytes allowed the extraction of patterns and the reconstruction of cortical areas inserted within spontaneous activity as the functional connectivity maps for the individual mice (O’Hashi et al., 2019). Confined, asynchronous, and spontaneous Ca^2+^ signals are commonly seen in fine astrocyte processes. As astrocytes are key elements regulating brain energy metabolism (Oheim et al., 2018), the way molecules signal and exchange metabolites in the neuron–glia–vascular circuitry and the concept of Ca^2+^ waves on microdomains will be greatly improved under the combination of powerful methodologies of calcium recording with fMRI.

One could also apply diffusion tensor imaging measurements, especially fractional anisotropy, with fMRI–BOLD responses to obtain coupled microstructural and functional imaging data (Warbrick et al., 2017). Data could be correlated, *a posteriori*, with chronic multi-site live calcium fluorescence for spatiotemporal measurements of neural activity. The knowledge acquired from such efforts would certainly be useful to corroborate molecular and histological results, strengthening the mechanistic inferences extracted from data.

Different mechanisms approaching external (channels, receptors, transporters, and exchangers) or internal (endoplasmic reticulum, calciosomes, vesicles) sources, using multiple steps and messengers/signaling molecules/binding proteins, virtually in every *in vitro* or *in vivo* cell, can be studied, and due to the scope and the space limitations of this review, we can only exemplify a few. Data shown are on the central and peripheral nervous system (retina and dorsal root ganglia and connections), neurons and glia population, glioblastoma, and cell lineages derived from human organoids.

## Retina, a Central Model

The retina is frequently chosen as a model to study interactions between neural cells due to its unique disposition in layers, from the embryonic to the final mature structure; as the retina develops, the environment changes, so extrinsic factors (transmitters, growth factors, extracellular matrix, and other mediators) interact with the genetic program modifying intrinsic transcription factors, so each cell leaves the cycle chronologically defined (Hatakeyama and Kageyama, 2004); retinal progenitors are multipotential, and region cell–cell interactions imply precise differentiation in a way that first retinal ganglion cells (RGCs), cone, and amacrine emerge from early-born progenitors, and then photoreceptors, bipolar, and Müller glia develop from late-born progenitors (Turner and Cepko, 1987). The vertical or radial axis, made by photoreceptors–bipolar–ganglion cells, is excitatory, while the horizontal or lateral axis, composed by horizontal and amacrine cells, is largely inhibitory (Barnstable, 1993). Photoreceptors release glutamate in the dark, which activates calcium-permeable ionotropic AMPA/NMDA receptors on OFF-type bipolar cells, while the activation of metabotropic glutamatergic type 6 receptor and ionotropic receptors, among others, mediates ON-type bipolar cell activity (Yang, 2004). Ca^2+^ imaging studies have been used to detail network properties in *ex vivo* retinal preparations and revealed a variety of responses on bipolar cells (at least eight different clusters) in terms of Ca^2+^ shifts in the axon terminals. On the other hand, activation of glutamatergic extrasynaptic NMDA receptors present in the rod pathway show increases in Ca^2+^ dendritic levels in both AII and A17 amacrine cells, postsynaptically at rod bipolar dyad synapses (Veruki et al., 2019). The depolarization of ganglion cells by voltage-dependent calcium channels (VDCC) contributes to soma and dendritic Ca^2+^ increases (Sargoy et al., 2014). These are a few examples on how live calcium imaging details the complex retinal signaling network. Based on two-photon calcium imaging, clustering of more than 11,000 mice RGC recordings shows that more than 30 functional channels are channeled (Baden et al., 2016). Recently, chronically stable *in vivo* recordings from RGCs in awake mice were reported using a genuine epiretinal-implanted 16-channel mesh electronic probe (Hong et al., 2018) delivered non-surgical intravitreally using syringe-injectable. Orientation-selective RGCs were stably registered between recording sessions, 7 days apart, implicating that individual cells can be tracked for 2 weeks.

Retinal neural cells (neurons, glia, and progenitors) can be phenotypically identified when matched in terms of calcium imaging (De Melo Reis et al., 2011). Progenitors cells usually express the intermediate filament marker nestin and are activated by muscimol, a GABA_*A*_ receptor-channel agonists in immature networks. The electrochemical gradient for Cl^–^ favors activation due to high intracellular Cl^–^ levels; in this sense, the expression of K^+^-coupled Cl^–^ transporter KCC2 is correlated with the modification of GABAergic transmission, switching during development from excitation on progenitors to inhibition on mature GABAergic neurons (Ganguly et al., 2001).

The neuron–glia circuits in the developing retina are shaped by light and sensed by intrinsically photon-detecting retinal ganglion cells, which are coupled electrically to other ganglion cells in a network (Sekaran et al., 2003). Beyond phototransduction, cones and rods regulate important physiological activities (circadian cycle, pupillary reflex, light sensing), which are frequently dependent on dopamine signaling (Caval-Holme et al., 2019). The dopaminergic system is one of the first phenotypes to be expressed in the developing retina (Reis et al., 2007), and dopamine is a potent modulator of spontaneous neural activity and gap junction coupling. Markers for young (doublecortin, β_*III*_-tubulin, and PSA-NCAM) and mature (MAP-2) retinal neurons are associated with KCl depolarizing cells (due to the expression of VDCC) [reviewed by Catterall (2000)]; retinal neurons are activated by ionotropic glutamatergic receptors, NMDA (Veruki et al., 2019), AMPA, or kainate (Okada et al., 1999; De Melo Reis et al., 2011; Passos et al., 2019). Some of these responses are modulated by cyclic AMP, which is induced by dopamine, adenosine, and PACAP receptors and functions as a differentiating factor for dopaminergic retinal cells in the chick (Guimaraes et al., 2001) or rat retina (Varella et al., 1999). Cannabinoid receptors present on neurons and glia (Kubrusly et al., 2018) on the avian retina also contribute to the regulation of different outputs such as GABA release, calcium entry, and cAMP mobilization.

**FIGURE 2:**
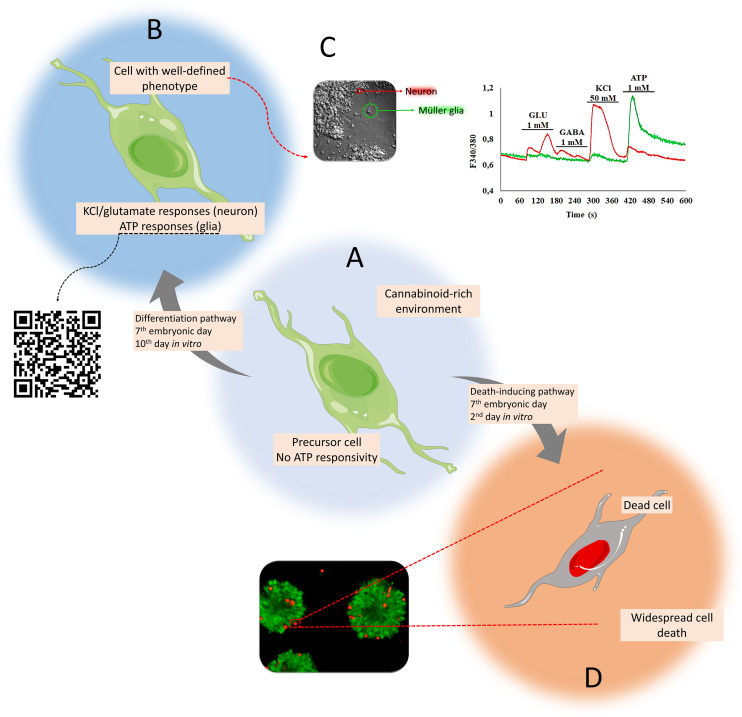
Matching cell phenotypes with calcium signaling activated by P2X7 receptors in retinal cells in culture. Progenitors unresponsive to ATP **(A)** differentiate into neurons (activated by KCl or glutamate) or glia (activated by ATP) in a cannabinoid-rich environment **(B)**. Emerging calcium shifts induced by ATP are exclusive to Müller glia (green trace), primed by the early cannabinoid receptor activation (with WIN 55,212-2). On the other hand, neurons are activated by KCl and glutamate (red trace) (Freitas et al., 2019) **(C)**. Instead of differentiating, some progenitors die **(D)** due to the activation of death-inducing pathways [bottom panel: live (calcein, green) and dead (ethidium homodimer-1, red)].

Müller cells are the main glial element in the retina, which intermingles with most, if not all, neurons in this tissue. These cells are active players (de Melo Reis et al., 2008) that transverse from the inner to the outer limiting membranes, spanning the whole retinal length, and control numerous activities such as osmotic (solute and water composition in the extracellular space), metabolic (glutamate-glutamine cycle), signaling (trophic factors and mediators), synaptic (release of transmitters), regulation of plasticity (Reichenbach and Bringmann, 2013), and response to excitotoxicity (Schitine et al., 2015). Müller cells are categorized by the expression of glutamine synthetase and glial fibrillary acidic protein, among other markers, and are activated by high concentrations of ATP due to the expression of purinergic calcium-permeable P2X7 receptor-channel, among others (De Melo Reis et al., 2011; Faria et al., 2017). ATP has many signaling and metabolic functions in the retina; it induces ionic waves through gap junctions and is involved with neuronal cell death (Metea and Newman, 2006; Reichenbach and Bringmann, 2016), while metabotropic (Keirstead and Miller, 1997) or ionotropic glutamatergic receptors on Müller glia also contribute to waves that depend on the nature of other transmitters involved (Rosa et al., 2015). Recent data have shown that progenitors from the embryonic avian retina unresponsive to ATP (Figure 2A) differentiate into neurons (activated by KCl or glutamate) or glia (activated by ATP) in a cannabinoid-rich environment (Figure 2B). Calcium shifts induced by ATP in avian Müller glia increased up to 30% in early retinal cells when cannabinoid receptors CB1 and CB2 are activated by the cannabinoid agonist WIN 55,212-2 (Figure 2C) (Freitas et al., 2019). Alternatively, a decrease in the number of glutamate-, GABA, and KCl-responsive cells (neurons and progenitors) was reported. Instead of differentiating, some progenitors die (D) due to the activation of death-inducing pathways (Figure 2D).

Müller cells control oxidative species as they participate in glutathione (GSH) synthesis as astrocytes do in the cortex. GSH is the main glial antioxidant found at a greater millimolar concentration when compared to neurons, which in turn show a much higher (10:1) ascorbate concentration compared to glia (Rice and Russo-Menna, 1998). GSH has been shown to induce selective Ca^2+^ shifts in Müller glia in a P2X7-dependent manner, a process that induces GABA release (Freitas et al., 2016), possible to strengthen retinal neuroprotection (Freitas and Reis, 2017). Interestingly, in a recent study where the P2X7 receptor was deleted, the basal GSH levels were decreased without altering GSH synthetic enzyme expression in the mouse hippocampus, a process linked to the glutamate–glutamine cycle and neutral amino acid transporters (Park and Kim, 2020).

The Müller glia has been gathering attention due to its proliferative properties after retinal injury and activation of stem/progenitor cell phenotype, allowing a return to the cell cycle and eventually differentiating into functional neurons. This has been shown for neurogenin 2 or ASCL1, transcriptional factors that, when nucleofected onto Müller glia, were reprogrammed into inducible neurons (Guimaraes et al., 2018), functionally monitored in terms of calcium imaging. This strategy has been used by many groups to partially restore vision in different animal models (Hampton, 2018).

Retinal information flows to the thalamus and the primary visual cortex (V1). Pyramidal cells in layers 2/3 were evaluated through two-photon Ca^2+^ imaging in GECI-expressing transgenic mice so excitatory and inhibitory neuron synchronization could be studied during the wakefulness of the animals (Knoblich et al., 2019). Calcium imaging data combined to *in vivo* targeted electrophysiology gave within-type cross-correlation coefficient (CC) profiles, which were used to characterize different populations of interneurons, parvalbumin, and VIP characterized as highly active and homogeneously synchronized; alternatively, somatostatin interneurons were subdivided into two populations, one spontaneously active, but uncorrelated to neighbor cells (Knoblich et al., 2019). These populations were differently modulated by locomotion, adding a functional perspective to neuronal cell typing.

## Subventricular Zone

The subventricular zone (SVZ) is considered as one of the main neurogenic niches in the brain (together with the subgranular zone), where the continuous proliferation of stem cells is actively balanced to generate progenitors, neurons, astrocytes, and oligodendrocytes (Eiriz et al., 2011). Each type of cell can be phenotypically identified based on selective markers associated with distinct mechanisms of Ca^2+^ entry, i.e., dependent on VDCC fast depolarization induced by KCl on neuroblasts and neurons (Schitine et al., 2012), coupled to metabotropic histamine receptors on progenitors, or modulated by thrombin through the protease-activating receptor on oligodendrocytes (Xapelli et al., 2013, 2014).

## Sensorial Stimuli, From DRG to Central Projections

Somatosensorial information combine different modalities such as pain, heat, touch, proprioception, and others by stimulation of peripheral receptors in pseudo-unipolar myelinated and unmyelinated first-order dorsal root ganglia (DRG) neurons; upon sensorial transduction, neural codes are generated as action potential patterns to cell bodies and then to synapses onto secondary neurons located in well-organized Rexed laminae of the spinal cord dorsal horn [reviewed in Peirs and Seal (2016)]. Parallel fibers carrying fast (*A*_*alpha*_/*A*_*beta*_, touch, and proprioception) or slow (*A*_*delta*_/*C*, temperature, and pain) submodalities flow to the thalamus and then to several brain areas.

Locally, the transient receptor potential acts as a non-selective cation channel family, whose vanilloid member (TRPV) depolarizes unmyelinated sensory neurons in the presence of capsaicin to temperature and painful thermal stimuli (Caterina et al., 1997; Peirs and Seal, 2016). Alternatively, ASIC1, acid-sensing ion channel 1, a member of the ASIC family of proteins and part of the degenerin/epithelial sodium channel (DEG/ENaC) superfamily, is expressed in large sensory myelinated neurons (Peirs and Seal, 2016).

**FIGURE 3:**
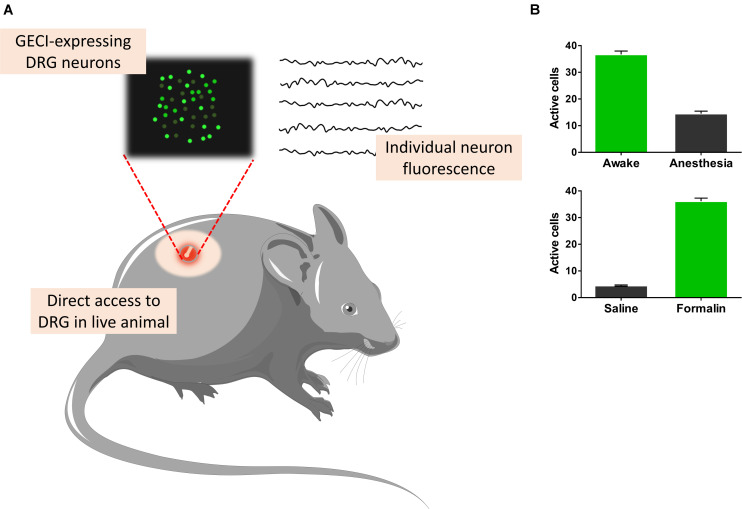
Recording dorsal root ganglia (DRG) cells from awake freely moving animals. Genetically coded calcium indicators (GECIs), which sense the concentration of Ca^2+^, allow for measurements of different cell types **(A)**. Freely behaving mice injected with plantar formalin showed DRG neuronal activity associated with phasic pain behavior (Chen et al., 2019). This activity persisted for 5 weeks as a hallmark of neuronal hyperactivity associated with ongoing pain (**B**, bottom). Alternatively, GECI-expressing transgenic mice allow the study of excitatory and inhibitory synchronization properties of anesthetized and awake (**B**, top) animals (Knoblich et al., 2019).

Calcium imaging has been employed to DRG and dorsal horn neuron co-cultures to understand the mechanisms behind synaptic transmission coded by *A*_*delta*_ (mechanical and thermal nociception) and C-fibers (high-intensity polymodal stimuli). It was shown that Ca^2+^ influx through NMDA receptors induced by glutamate depolarizes dorsal horn neurons (Ohshiro et al., 2007). Schwann and satellite cells interact closely with DRG neurons. At the cellular level, KCl and ATP activate selectively neurons and glia, respectively (Ribeiro-Resende et al., 2014). VDCC are expressed in postnatal primary sensory neurons, while purinergic receptors are broadly expressed in all major classes of glia, including Schwann cells. Indeed purinergic signaling controls many important functions in the neuro-glia compartment in the DRGs such as proliferation, motility, survival, differentiation, and myelination (Fields and Burnstock, 2006). The use of two-photon Ca^2+^ imaging, Ca^2+^ indicators, and/or GECI allow high-resolution measurements with detailed functions of different cell types in DRG, for instance, a particular group of neurons (11 to 33 μm in diameter) is activated by an increase in temperature in the animal hind paw (Anderson et al., 2018). Besides that, freely behaving mice injected with plantar formalin had a DRG neuronal activity (Figure 3) associated with phasic pain behavior (Chen et al., 2019). This activity persisted for 5 weeks as a hallmark of neuronal hyperactivity associated with ongoing pain. Classical cannabinoid G protein receptors (CB1 and CB2) have been studied in addition to TRPV1 in DRG neurons to evaluate how endocannabinoids can control pain (Millns et al., 2006). Ca^2+^ influx through purinergic, carbachol-activating muscarinic or TRPV1 receptors increases anandamide synthesis within cells (Ahluwalia et al., 2003) and activates both TRPV1 (van der Stelt et al., 2005) and cannabinoid receptors. CB1 receptors co-localized with purinergic receptors in DRG small-diameter neurons control pain by anandamide and involves the modulation of P2X3 receptor in the primary afferent neuron (Oliveira-Fusaro et al., 2017).

Neurons and glia (visualized as ramified processes and in contact with blood vessels, labeled as SR101+) were also studied in response to heat/cold stimuli in the superficial dorsal horn from anesthetized mice (Johannssen and Helmchen, 2010; Ran et al., 2016). The authors reported different overlapping neurons on dorsal horn ensembles activated by cutaneous stimulation in freely behaving mice, while it was reported that large-scale coordinated calcium responses were generated in astrocytes in response to intense, but not weak, sensory inputs (Sekiguchi et al., 2016). On the other hand, amygdala has also received attention as a hub in the pain matrix, as the basolateral part of the amygdala contributes to the negative affective perception of unpleasant pain (Corder et al., 2019), and P2X7 receptor acts in the modulation of neuropathic pain (Hu et al., 2020). Interestingly, the role of general anesthetics on calcium imaging in awake head-restrained mice was studied, and a combination of ketamine/xylazine, isoflurane, and urethane abolished calcium responses in neocortical astrocytes, modifying synchronized widespread transients related to arousal in awake animals (Thrane et al., 2012). Effects were associated with inositol 1,4,5-triphosphate type 2 receptor but independent of glutamatergic or purinergic signaling.

Microglia, the immune resident cells in the nervous system, and monocyte-derived macrophages derived from the peripheral circulation have significant and distinct roles in neuroinflammation, injured spinal cord (David and Kroner, 2011), or/and progression of neurological and neuropsychiatric diseases (Chen et al., 2018; Tvrdik et al., 2019). Spinal cord lesion is associated with secondary network damage through inflammation, ischemia, edema, generation of reactive oxygen species, and excitotoxicity (Kroner and Rosas Almanza, 2019). Calcium shifts activated by purines, through P2X receptors, particularly P2X7 and P2X4, mediate neuropathic pain. Indeed activation of microglia by ATP contributes to tactile allodynia in the spinal cord (Tsuda et al., 2003), and microglia–neuron interactions are greatly affected in male, but not female, mice, caused by peripheral nerve injury. Inhibition of purinergic P2X4 receptors decreased pain hypersensitivity in male rats only (Mapplebeck et al., 2018). The microglia activated by ATP also releases brain-derived neurotrophic factor (BDNF) on lamina I neurons that change the polarity of currents activated by GABA, a major effect after peripheral nerve injury (Coull et al., 2003). As BDNF is an important player between microglia and neurons, this might represent a therapeutic strategy for treating neuropathic pain (Coull et al., 2005).

Generation of conditional mouse reporter of calcium facilitated the deployment of GECI in microglia, permitting *in vivo* studies of intracellular calcium in large microglial cell populations in cerebral pathologies such as ischemic stroke. The use of reporter mice and recent GECI indicated novel roles in development and plasticity-driven reorganization, as well as a surveillant cell in the homeostatic maintenance of brain tissue (Brawek and Garaschuk, 2013); large cell populations have been imaged in ischemic stroke *in vivo* (Tvrdik et al., 2019).

One of the hottest topics in the last decade related to microglia is the role in neural circuits refinement *via* both promoting synapse formation and by targeting specific synapses for elimination and phagocytosis (Akiyoshi et al., 2018). Ca^2+^ imaging of larger populations of motor cortical layer 2/3 pyramidal neurons show microglia–dendritic spine interactions that increase neuronal activity and consequent neuronal synchronization in healthy brain in awake mice (Akiyoshi et al., 2018).

## Cancer

### Glioblastoma as a Model

Cancer affects millions worldwide and comprises a multifaceted pathology of different histopathological types. The cancer microenvironment has a unique scenario and shares common strategies for development. Therefore, the use of a methodology to selectively identify, differentiate, and gather information through a functional imaging property is highly desirable.

**FIGURE 4:**
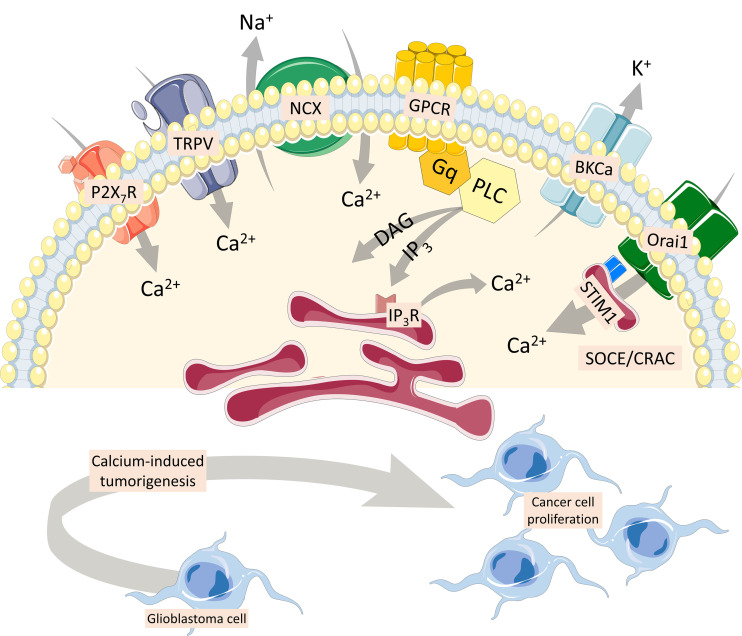
Multiple Ca^2+^ pathways involved in cancer cells. P2X7 and TRPV1 receptors activate a strong calcium response in cancer cells (Strong and Daniels, 2017). Glutamate secretion upon ATP activation is excitotoxic to healthy tissue and linked to tumorigenicity into adjacent brain regions (Strong et al., 2018). The Na^+^/Ca^2+^ exchanger (NCX), which maintains cytoplasmic calcium homeostatic levels, also increases Ca^2+^ levels in glioblastoma cells (Song et al., 2014). Inhibition of the forward NCX (Ca^2+^ exit mode) induces a Ca^2+^-mediated injury in glioblastoma cells (Hu et al., 2019). Finally, G protein-coupled metabotropic receptors coupled to activation of phospholipase C result in the generation of the second messenger, inositol 1,4,5 trisphosphate, which increases intracellular Ca^2+^ and diacylglycerol, involved in cancer cell proliferation, with important participation of the large-conductance voltage- and Ca^2+^-activated K^+^ channel. Store-operated Ca^2+^ entry is mediated through Ca^2+^ release-activated Ca^2+^ current, composed by ORAI1, and stromal interaction molecule 1 (STIM-1), a luminal Ca^2+^ sensor transmembrane protein present in the endoplasmic reticulum membrane (Putney, 2009; De Bock et al., 2013). Both ORAI1 and STIM-1 are upregulated in primary human cell lines obtained from samples of glioblastoma (Motiani et al., 2013).

Glioblastoma multiforme is a grade IV malignant, astrocyte-derived brain tumor, highly heterogeneous, with unfavorable prognosis. Seizures are frequently seen among patients due to an imbalance in the inhibitory interneuron network and excess of glutamate release (Hatcher et al., 2020). In this sense, cell lines have been used to model glioblastoma and other high-grade gliomas, and calcium imaging has been applied as a strategy to evaluate receptors/transporters/exchangers from tumors and healthy cells. GL261 cell line has been used to match pharmacological treatments based on cellular phenotype, cultured under adherent or non-adherent conditions. Both ATP (through P2X7 receptor) and capsaicin (TRPV1 receptor) activate a strong calcium response in GL261 neurospheres, but not on adherent cells (Strong and Daniels, 2017). This cell line secretes glutamate upon ATP activation, which is excitotoxic to healthy tissue and linked to tumorigenicity into adjacent brain regions (Strong et al., 2018). In addition to P2X7 and TPRV receptors, the Na^+^/Ca^2+^ exchanger (NCX), when activated by SKF 96365 (TRPC channel blocker), increases Ca^2+^ levels in glioblastoma cells (Figure 4). NCX controls intracellular Ca^2+^ homeostasis, and silencing of NCX1 isoforms diminished the effect of SKF 96365 on glioblastoma cells (Song et al., 2014). In addition, inhibition of the forward NCX (Ca^2+^ exit mode), with bepridil and CB-DMB, induced Ca^2+^-mediated injury in glioblastoma cells (Hu et al., 2019). NCX maintains cytoplasmic calcium homeostatic levels, in addition to plasma membrane Ca^2+^ transport ATPase (PMCA) and sarco/endoplasmic reticulum (SR/ER) Ca^2+^-ATPase (SERCA).

The P2X7 receptor is found in several types of tumors, such as neuroblastoma, melanoma, prostate, lung, and breast cancer. P2X7 expression is commonly associated with cancer cell survival, proliferation, and metastatic potential; upon dysregulation, the P2X7 receptor is associated with tumor initiation and development (Gilbert et al., 2019).

ING5, a member of the ING family of epigenetic regulators that alter histone acetylation and, subsequently, gene expression, increased the expression of stem cell markers and, consequently, brain tumor-initiating cells that cause the recurrence of glioblastomas (stemness) affected by epigenetic mechanisms (Wang F. et al., 2018). Proteomics and cell imaging assays demonstrated that ING5-transfected cells had intracellular Ca^2+^ elevation, which was associated with self-renewal, due to an increased expression of several types of Ca^2+^ channels, like L-type and P/Q-type voltage-gated Ca^2+^ channels, and TRPC (Wang F. et al., 2018). Differentiated glioblastoma cells constitute a distinctive niche compared to glioblastoma stem cells, sending cues to the microenvironment, both as juxtacrine (involving direct contact) or paracrine (through secreted factors). One of these is BDNF, secreted by SOX2-negative tumor cells (Wang X. et al., 2018). Activation of NTRK2, the cognate receptor, activates the PI3K-AKT pathway which is activated in glioblastoma stem cells, leading to tumor growth (Wang X. et al., 2018).

Store-operated Ca^2+^ entry (SOCE) is mediated through Ca^2+^ release-activated Ca^2+^ current (CRAC), non-voltage sensitive channels highly selective for Ca^2+^. They are composed by ORAI1, a member of the ORAI Ca^2+^ channel family (also known as CRAC modulator 1 or CRACM1), and form a macromolecular complex with the stromal interaction molecule 1 (STIM-1), a luminal Ca^2+^ sensor transmembrane protein present in the ER membrane (Putney, 2009; De Bock et al., 2013). Both ORAI1 and STIM-1 are upregulated in primary human cell lines obtained from surgical samples of glioblastoma multiforme compared to non-tumor human astrocytes (Motiani et al., 2013). CRAC inhibitors SKF-96365, 2-APB, and diethylstilbestrol also blocked GBM cell proliferation, and silencing of ORAI1 and STIM1 proteins using siRNA significantly inhibited C6 cell proliferation and SOCE compared to control cells (Liu et al., 2011).

Mice generated with CRISPR-based *in utero* electroporation of three deleted genes encoding phosphatase and tensin homolog (Pten), neurofibromin 1 (Nf1), and p53 (Trp53), all linked to tumorigenesis in human glioma genes, show a brain tumor with progressive cortical hyperexcitability due to tumor invasion and spontaneous seizures. Iba1^+^ cells (microglia) also increased fivefold in the neighboring tumor area. Intracellular calcium imaging revealed recurrent seizure activity and robust bilateral calcium activity in GCAMP unanesthetized mice (Hatcher et al., 2020).

Metabotropic receptors coupled to the activation of phospholipase C (PLC) results in phosphatidylinositol 4,5 biphosphate cleavage and generation of the second messengers inositol 1,4,5 trisphosphate (IP3), which increases intracellular Ca^2+^, and diacylglycerol, which activates protein kinase C. Activation of IP3 receptors on intracellular stores (endoplasmic reticulum and calciosomes) permits the release of Ca^2+^ down its electrochemical gradient, reaching cytoplasmic levels up to 10^–6^ M (Berridge et al., 2000). Type 3 IP3 receptor (IP3R3) and voltage- and Ca^2+^ dependent K(+) channels (BKCa) were shown to participate in breast cancer cell proliferation. Cell lines MCF-7 and MCF-10A are activated by 25 μM ATP, which induces a PLC-dependent elevation of intracellular Ca^2+^ and cell proliferation, which is ablated when the expression of both BKCa and/or IP3R3 is inhibited by specific small interfering RNAs, leading to a cell cycle arrest in the G0/G1 phase (Mound et al., 2013). Alternatively, live-cell imaging of intracellular calcium fluxes has been used in human parathyroid tumor sections upon calcium-sensing receptor activation, a metabotropic receptor, and consequently visualize the effects of intratumoral heterogeneity in real time (Koh et al., 2017).

## Neural Cell Lineages Derived From iPS Cells

Induced pluripotent stem (iPS) cells have been generated from mouse and human somatic cells by introducing selective transcription factors (Oct3/4, Sox2, Klf4, c-Myc, Nanog, and/or Lin28) using retroviruses or lentiviruses (Okita et al., 2008). Differentiation of iPS on neural cells from self-organized human organoids/spheroids has recently been used to generate multiple classes of cells: progenitors, neurons, and glia to be enriched and considered to be used in healthy and disease models, such as neurological disorders. Indeed astrocyte (Dezonne et al., 2017; Sloan et al., 2017), oligodendrocyte (Marton et al., 2019) and neuron (Nascimento et al., 2019) lineage cells, as well as region-specific cortical neurons and astrocytes, some from both deep and superficial cortical layers that are transcriptionally correlated to *in vivo* fetal development (Paşca et al., 2015), have been generated following multiple strategies, of which functionality data included calcium imaging assays. Derived glia from human cerebral cortical spheroids had similar properties found in primary fetal astrocytes, and upon transition to a more developed state, dozens of astrocytic markers increased as expected, accelerating around birth, while fetal astrocyte markers declined rapidly over this same period. Gene analysis can be hierarchically organized and disposed as three primary clusters of cells: for instance, the expression levels of aquaporin-4, the transcription factor SOX9, and the glial high-affinity glutamate transporter were compatible to astrocyte lineage cells, but not neurons derived from spheroids (Sloan et al., 2017); changes in calcium signaling, phagocytic capacity, and transcriptional regulation approached to those observed in a mature state (Sloan et al., 2017). In general, emerging cortical organoids from iPS replicates most of the characteristics found in the *in vivo* cortex, including temporal corticogenesis and connectivity. The exception seems to be generation of microglia and blood vessels (Varrault et al., 2019). Co-cultures of astrocytes generated from human cerebral cortical spheroids in the presence of early *in vitro*-stage neurons produced an increase in depolarization-induced calcium signaling (Sloan et al., 2017).

Electrophysiology is also a valuable tool to evaluate organoids at the molecular and the cellular levels, comparing gene expression profiles among fetal and adult brains using cerebral organoids (Logan et al., 2020). The activity of cultured neurons from human dissociated embryonic stem cell-derived cerebral organoids was also evaluated through calcium imaging, and the activities were shown to follow synchronized and non-synchronized patterns (Sakaguchi et al., 2019). Part of this activity depends on gamma-aminobutyric acid (GABA) and gap junctions to the development of synchronous activity in hPSC-derived neural networks (Mäkinen et al., 2018). Human organoids are also being developed to model glioblastoma, so invasion can be studied and how tumorigenesis relates to the extracellular matrix can be understood (Silvia and Dai, 2020). Tumor cells within organoids extend a network of long microtubes, recapitulating the *in vivo* behavior of GBM (Krieger et al., 2020). The transcriptional changes implicated in the invasion process are coherent across patient samples, indicating that GBM cells reactively upregulate genes required for their dispersion.

Millions of chronic disease patients with organ insufficiency that cannot be treated with transplant or pharmacotherapy rely on the possibility that 1 day personalized-specific iPS cells might integrate in target tissues and become a routine in regenerative medicine. In terms of brain function, this type of approach is being used to treat ischemia to replenish neural cells and to approach autism spectral disorder as many proteins involved in the regulation of synaptic plasticity, neuronal excitability, and neuronal connectivity are inappropriately developmentally generated or misplaced, disturbing the neuronal network activity (Culotta and Penzes, 2020). On Alzheimer’s disease, early hyperexcitability is linked to widespread synapse loss and cognitive dysfunction, and therefore a culture model derived from human cerebral organoids might shed some light on a synaptic plasticity model comparing aberrant neural networks from controls (Ghatak et al., 2020).

## Final Remarks

Calcium imaging has been used in diverse dynamic cellular networks to correlate spatiotemporal calcium shifts to different intracellular and extracellular mechanisms. The transition of progenitors and stem cells to differentiated neurons, astrocytes, oligodendrocytes, and microglia can be followed through ion channels, transporters, and receptors coupled to calcium fluxes. Cancer cells or inducible pluripotent cell lines might help to model diseases and can be differentiated from healthy cells in terms of the expression of calcium-binding/permeable molecules and shed light on therapy.

## Author Contributions

RM, HF, and FM contributed to the review body text and the conception of the figures. All authors contributed to the article and approved the submitted version.

## Conflict of Interest

The authors declare that the research was conducted in the absence of any commercial or financial relationships that could be construed as a potential conflict of interest.
